# Supplementary material for: Characteristics, source apportionment and health risks of indoor and outdoor fine particle-bound polycyclic aromatic hydrocarbons in Jinan, North China
Source: PeerJ. 2024 Dec 16;12:e18553. doi: 10.7717/peerj.18553 (PMC11657193; doi:10.7717/peerj.18553)
Supplement: Supplemental Information 2 [file peerj-12-18553-s002.docx]

***Supplementary Material***

**Characteristics, source apportionment and health risks of indoor and outdoor fine particle-bound polycyclic aromatic hydrocarbons in Jinan, North China**

Xiaomei Gao^1^, Ziyi Wang^2^, Xiaoyan Sun^3^, Weidong Gao^1^, Wei Jiang^1^, Xi Wang^1^, Fenfen Zhang^1^, Xinfeng Wang^2^, Lingxiao Yang^2^, Yang Zhou^4,5^

^1^ School of Water Conservancy and Environment, University of Jinan, Jinan, Shandong Province, China

^2^ Environment Research Institute, Shandong University, Qingdao, Shandong Province, China

^3^ Jinan Ecological and Environmental Monitoring Center, Jinan, Shandong Province, China

^4^ Frontier Science Center for Deep Ocean Multispheres and Earth System (FDOMES) and Physical Oceanography Laboratory, Ocean University of China, Qingdao, Shandong Province, China

^5^ College of Oceanic and Atmospheric Sciences, Ocean University of China, Qingdao, Shandong Province, China

Corresponding Author:

Weidong Gao^1^

University of Jinan, Nanxinzhuang West Road, Jinan, Shandong Province, 250022, China

Email address: stu_gaowd@ujn.edu.cn

**List of the supplementary information:**

**Figures S1 to S7**

Figure S1. Location of the sampling site (a), and the picture of the vacant office (b).

Figure S2. Chromatogram of the chromatography-single quadrupole mass spectrometry.

Figure S3. Rotating space component diagram from PCA analysis.

**Tables S1 to S3**

Table S1. TEF_i_ and RfD_i_ values of individual PAHs.

Table S2. Parameters used in incremental lifetime cancer risk assessment.

Table S3. Comparison of the measured outdoor PM_2.5_-bound PAHs at different sites (ng/m^3^).

Table S4. Comparison of the measured indoor and outdoor PM_2.5_-bound PAHs at different sites (ng/m^3^).

Table S5. Indoor/outdoor relationships of PAHs.

Table S6. The correlation between PAHs with temperature and wind speed.

Table S7. TEQ_Bap_ values (ng/m^3^) calculated from PAHs.

Table S8. HI values of PAHs by aged-specific groups.

Table S9. ILCR values of PAHs by aged-specific groups.

**Figure S1** Location of the sampling site (a), and the picture of the vacant office (b).


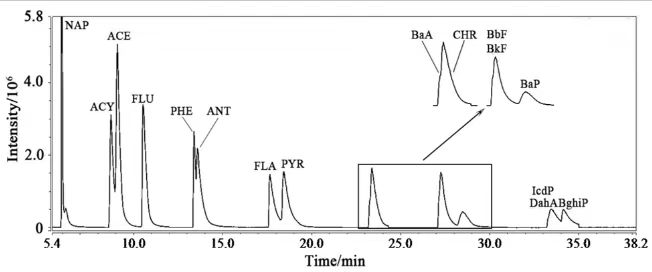


**Figure S2** Chromatogram of the chromatography-single quadrupole mass spectrometry.


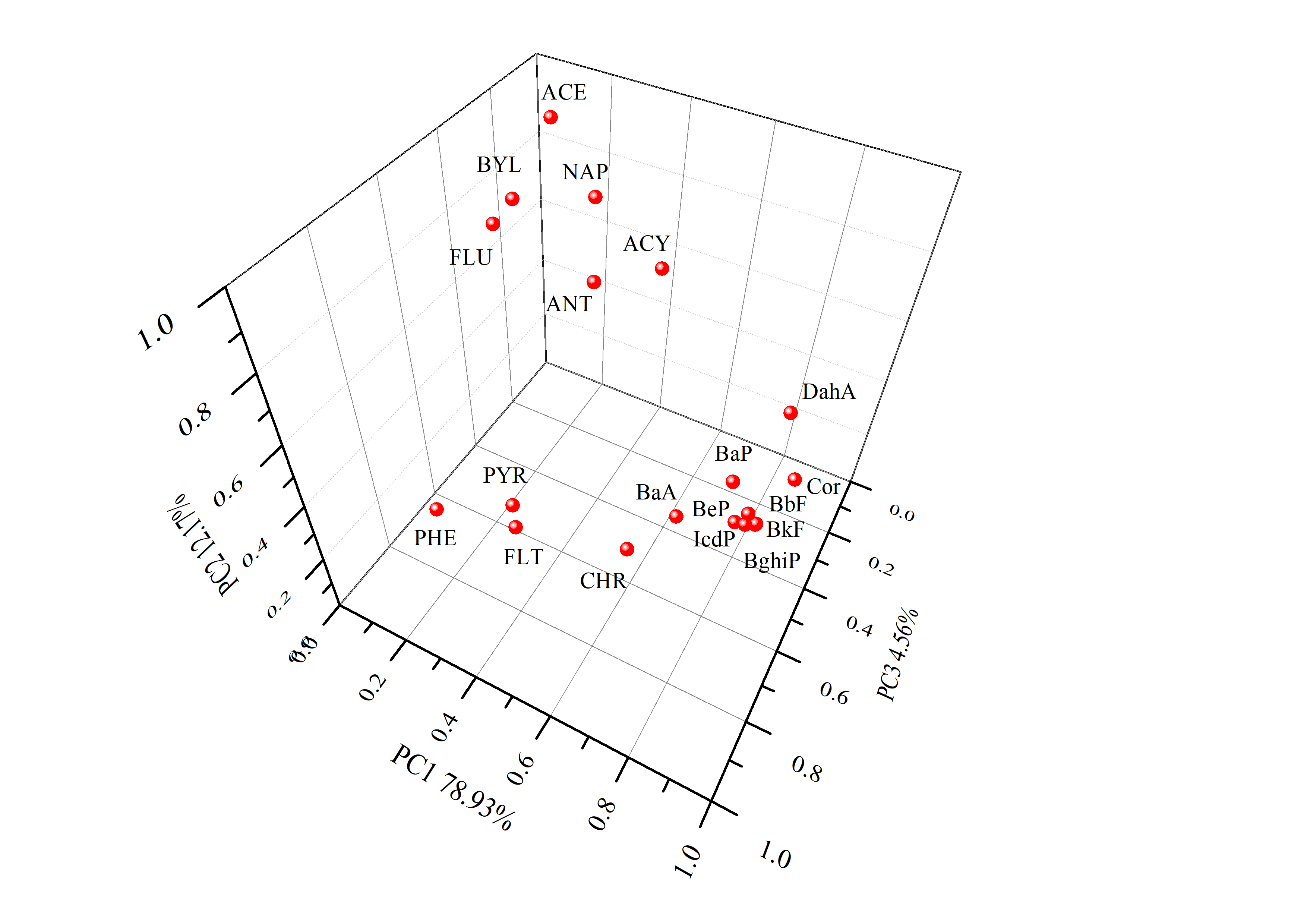


**Figure S3** Rotating space component diagram from PCA analysis.

**Table S1** TEF_i_ and RfD_i_ values of individual PAHs.

| Species | TEF_i_ | RfD_i_ |
| --- | --- | --- |
| NAP | 0.001 | 0.02 |
| ACY | 0.001 |  |
| ACE | 0.001 | 0.06 |
| FLU | 0.001 | 0.04 |
| PHE | 0.001 | 0.015 |
| ANT | 0.01 | 0.3 |
| FLT | 0.001 | 0.04 |
| PYR | 0.001 | 0.015 |
| BaA | 0.1 |  |
| CHR | 0.01 |  |
| BkF | 0.1 |  |
| BbF | 0.1 |  |
| BeP | 0.002 |  |
| BaP | 1 | 0.0003 |
| DahA | 1 |  |
| IcdP | 0.1 |  |
| BghiP | 0.01 | 0.015 |

**Table S2** Parameters used in incremental lifetime cancer risk assessment.

| Parameter | Unit | Childhood (1-11) | | Adolescence (12-17) | | Adulthood (18-70) | |
| --- | --- | --- | --- | --- | --- | --- | --- |
|  |  | Male | Female | Male | Female | Male | Female |
| BW | kg | 17.2 | 16.5 | 47.1 | 44.8 | 71.4 | 63 |
| CSF_Inhalation_ | (mg/(kg·d))^-1^ | 3.85 | 3.85 | 3.85 | 3.85 | 3.85 | 3.85 |
| CSF_Infestion_ | (mg/(kg·d))^-1^ | 7.3 | 7.3 | 7.3 | 7.3 | 7.3 | 7.3 |
| CSF_Dermal_ | (mg/(kg·d))^-1^ | 25 | 25 | 25 | 25 | 25 | 25 |
| IR_Inhalation_ | m^3^/d | 8.4 | 8.4 | 13.1 | 13.1 | 18.7 | 15.1 |
| IR_Ingestion_ | mg/d | 200 | 200 | 200 | 200 | 100 | 100 |
| ED | year | 6 | 6 | 14 | 14 | 30 | 30 |
| AT | d | 25550 | 25550 | 25550 | 25550 | 25550 | 25550 |
| SA | cm^2^/d | 2800 | 2800 | 2800 | 2800 | 5700 | 5700 |
| AF | mg/cm^2^ | 0.2 | 0.2 | 0.2 | 0.2 | 0.07 | 0.07 |
| ABS |  | 0.13 | 0.13 | 0.13 | 0.13 | 0.13 | 0.13 |
| PEF | m^3^/kg | 1.36E+09 | 1.36E+09 | 1.36E+09 | 1.36E+09 | 1.36E+09 | 1.36E+09 |

**Table S3** Comparison of the measured outdoor PM_2.5_-bound PAHs at different sites (ng/m^3^).

| Site | Date | Species | Outdoor | References |
| --- | --- | --- | --- | --- |
| **Jinan, urban**  **Jinan, urban**  Jinan, urban | **June 21 to July 5, 2019**  **October 26 to November 13, 2019**  June 22 to July 04, 2015 | **19**  **19**  17 | **11.05**  **32.99**  19.45 | **This study**  **This study**  Zhang et al., 2018 |
| Jinan, urban | June 3 to 15, 2016 | 18 | 14.3 | Zhang et al., 2019a |
| Jinan, urban | September 20 to 29, 2016 | 18 | 21.9 | Zhang et al., 2019a |
| Jinan, urban | 17 July to 26 July, 2010 | 15 | 30.78 | Zhu et al., 2015 |
| Jinan, urban | 11 October to 27 October, 2010 | 15 | 140.34 | Zhu et al., 2015 |
| Jinan, urban | January 6 to January 29, 2016 | 19 | 105.3 | Li et al., 2017 |
| Beijing, urban | July, 2017 | 16 | 5.58 | Li et al., 2022 |
| Beijing, urban | October, 2017 | 16 | 11.48 | Li et al., 2022 |
| Beijing, urban | 2015.7.16– 8.29 | 16 | 9.2 | Chen et al., 2017 |
| Beijing, urban | 2015.9.2– 9.29 | 16 | 15 | Chen et al., 2017 |
| Dongguan | summer, 2017-2018 | 16 | 0.77 | Chen et al., 2022 |
| Dongguan | winter, 2018-2018 | 16 | 12.72 | Chen et al., 2022 |
| Shanghai, urban | July and August, 2015 | 15 | 3.04 | Hong et al., 2021 |
| Shanghai, urban | November, 2014 | 15 | 15.7 | Hong et al., 2021 |
| Nanjing, urban | July and August, 2015 | 15 | 2.67 | Hong et al., 2021 |
| Nanjing, urban | November, 2014 | 15 | 21.69 | Hong et al., 2021 |
| Taipei, urban | January 23rd to February 25th,2021 | 16 | 0.96 | Ting et al., 2024 |
| Taipei, urban | June 3rd to August 29th,2021 | 16 | 0.43 | Ting et al., 2024 |
| Xi'an | summer, 2017 | 16 | 40.6 | Wang et al., 2019 |
| Xi'an | autumn, 2017 | 16 | 62.2 | Wang et al., 2019 |
| Harbin | summer, 2017 | 16 | 5.88 | Ma et al., 2020 |
| Harbin | autumn, 2017 | 16 | 72.1 | Ma et al., 2020 |
| Wuhan, urban | 12th Jun— 22th Jul, 2014 | 16 | 11.3 | Zhang et al., 2019b |
| Wuhan, urban | 8th Oct—6th Nov, 2014 | 16 | 23.5 | Zhang et al., 2019b |
| Caofeidian, suburban | 25 June to 4 August 2018 | 18 | 16.39 | Zhang et al., 2021 |
| Caofeidian, suburban | 3 September to 13 October 2018 | 18 | 25.22 | Zhang et al., 2021 |
| Rome, Italy, urban | Jan-Feb 2012 | 8 | 17.94 | Romagnoli et al., 2014 |
| Rome, Italy, urban | May-Jun 2012 | 8 | 0.62 | Romagnoli et al., 2014 |
| Rome, Italy, urban | Jan-Feb 2012 | 8 | 8.89 | Romagnoli et al., 2014 |
| Rome, Italy, urban | May-Jun 2012 | 8 | 1.27 | Romagnoli et al., 2014 |
| Kanazawa, Japan, urban | summer, 2016-2017 | 9 | 0.16 | Kalisa et al., 2019 |
| Kanazawa, Japan, urban | autumn, 2016-2017 | 9 | 0.89 | Kalisa et al., 2019 |
| Auckland, New Zealand, urban | summer, 2016-2017 | 9 | 0.18 | Kalisa et al., 2019 |
| Auckland, New Zealand, urban | autumn, 2016-2017 | 9 | 0.33 | Kalisa et al., 2019 |
| Tehran, Iran, urban | summer, 2018 | 16 | 26.1 | Ali-Taleshi et al., 2021 |
| Tehran, Iran, urban | autumn, 2018 | 16 | 37.6 | Ali-Taleshi et al., 2021 |

**Table S4** Comparison of the measured indoor and outdoor PM_2.5_-bound PAHs at different sites (ng/m^3^).

| Site | Periods | Species | Indoor | Outdoor | Indoor environmental type | References |
| --- | --- | --- | --- | --- | --- | --- |
| **Jinan** | **June 21 to July 5, 2019** | **19** | **9.92** | **11.05** | **Office** | **This study** |
| **Jinan** | **October 26 to November 13, 2019** | **19** | **24.82** | **32.99** | **Office** | **This study** |
| Jinan, urban | 17 July to 26 July, 2010 | 15 | 29.20 | 30.78 | Office | Zhu et al., 2015 |
| Jinan, urban | 11 October to 27 October, 2010 | 15 | 111.07 | 140.34 | Office | Zhu et al., 2015 |
| Jinan, urban | January 6 to January 29, 2016 | 19 | 63.26 | 105.3 | Hotel | Li et al., 2017 |
| Jinan, suburban | January 6 to January 29, 2016 | 19 | 39.58 | 67.96 | Hotel | Li et al., 2017 |
| Beijing, urban | July 16 to August 29, 2015 | 16 | 9.7 | 9.2 | Office | Chen et al., 2017 |
| Beijing, urban | September 2 to September 29, 2015 | 16 | ~15.0 | 15.0 | Office | Chen et al., 2017 |
| Beijing, urban | September 2 to September 29, 2015 | 16 | ~15.0 | 15.0 | Students' dormitory | Chen et al., 2017 |
| Beijing, urban | July 16 to August 29, 2015 | 16 | 12.3 | 9.2 | Residential apartment | Chen et al., 2017 |
| Beijing, urban | September 2 to September 29, 2015 | 16 | ~15.0 | 15.0 | Residential apartment | Chen et al., 2017 |
| Beijing, urban | September 15 to 22, 2015 | 11 | 8.75 | 8.95 | Classroom | Zhang et al., 2020 |
| Caofeidian, suburban | 25 June to 4 August 2018 | 18 | 15.96 | 16.39 | Students’ laboratory | Zhang et al., 2021 |
| Caofeidian, suburban | 3 September to 13 October 2018 | 18 | 21.17 | 25.22 | Students’ laboratory | Zhang et al., 2021 |
| Caofeidian, suburban | 12 April to 22 May 2018 | 18 | 18.15 | 20.25 | Students’ laboratory | Zhang et al., 2021 |
| Caofeidian, suburban | 7 November to 17 December 2018 | 18 | 44.23 | 189.63 | Students’ laboratory | Zhang et al., 2021 |
| Beijing, urban | December, 2014 to March, 2015 | 16 | 187.3 | 387.0 | Residential apartment | Han et al., 2016 |
| Rome, urban, Italy | Jan-Feb 2012 | 8 | 5.73 | 17.94 | Unused office | Romagnoli et al., 2014 |
| Rome, urban, Italy | May-Jun 2012 | 8 | 0.40 | 0.62 | Unused office | Romagnoli et al., 2014 |
| Rome, urban, Italy | Jan-Feb 2012 | 8 | 7.12 | 8.89 | Office | Romagnoli et al., 2014 |
| Rome, urban, Italy | May-Jun 2012 | 8 | 0.73 | 1.27 | Office | Romagnoli et al., 2014 |
| Dąbrowa Górnicza, urban, Poland | 10 April–3 May 2010 | 15 | 36.1 | 52.9 | Kindergartens | Błaszczyk et al., 2017 |
| Złoty Potok, rural, Poland | 17 March–09 April 2010 | 15 | 31.4 | 39.3 | Kindergartens | Błaszczyk et al., 2017 |

**Table S5** Indoor/outdoor relationships of PAHs.

|  | Overall (n=31) | |  | Summer (n=13) | |  | Autumn (n=18) | |
| --- | --- | --- | --- | --- | --- | --- | --- | --- |
|  | r | I/O |  | r | I/O |  | r | I/O |
| NAP | 0.69** | 0.97±0.21 |  | 0.75** | 1.00±0.21 |  | 0.62^**^ | 0.95±0.21 |
| BYL | 0.57** | 1.04±0.26 |  | 0.59* | 1.00±0.19 |  | 0.45 | 1.07±0.30 |
| ACY | 0.81** | 0.97±0.13 |  | 0.92** | 0.99±0.07 |  | 0.65^**^ | 0.95±0.16 |
| ACE | 0.55** | 0.99±0.19 |  | 0.73** | 0.96±0.12 |  | 0.41 | 1.01±0.23 |
| FLU | 0.67** | 0.97±0.17 |  | 0.85** | 0.98±0.15 |  | 0.45 | 0.96±0.19 |
| PHE | 0.83** | 0.99±0.31 |  | 0.77** | 1.10±0.35 |  | 0.39 | 0.90±0.24 |
| ANT | 0.65** | 0.97±0.17 |  | 0.82** | 1.07±0.11 |  | 0.49^*^ | 0.91±0.18 |
| FLT | 0.90** | 0.99±0.42 |  | 0.80** | 1.20±0.53 |  | 0.67^**^ | 0.83±0.23 |
| PYR | 0.85** | 0.95±0.37 |  | 0.77** | 1.12±0.43 |  | 0.54^*^ | 0.82±0.27 |
| BaA | 0.93** | 0.86±0.24 |  | 0.74** | 1.04±0.23 |  | 0.85^**^ | 0.72±0.15 |
| CHR | 0.90** | 0.80±0.31 |  | 0.74** | 0.97±0.38 |  | 0.80^**^ | 0.66±0.15 |
| BkF | 0.91** | 0.92±0.55 |  | 0.76** | 1.12±0.72 |  | 0.84^**^ | 0.78±0.35 |
| BbF | 0.90** | 0.86±0.28 |  | 0.80** | 0.93±0.27 |  | 0.85^**^ | 0.81±0.28 |
| BeP | 0.90** | 0.90±0.53 |  | 0.77** | 1.05±0.68 |  | 0.83^**^ | 0.79±0.37 |
| BaP | 0.91** | 0.86±0.31 |  | 0.82** | 0.86±0.22 |  | 0.85^**^ | 0.87±0.37 |
| DahA | 0.87** | 0.86±0.19 |  | 0.86* | 0.84±0.11 |  | 0.85^**^ | 0.87±0.22 |
| IcdP | 0.92** | 0.85±0.38 |  | 0.72** | 0.88±0.39 |  | 0.88^**^ | 0.83±0.39 |
| BghiP | 0.92** | 0.83±0.38 |  | 0.87** | 0.83±0.41 |  | 0.8^**^ | 0.83±0.38 |
| Cor | 0.92** | 0.86±0.26‘ |  | 0.81** | 0.86±0.16 |  | 0.89^**^ | 0.85±0.31 |
| LMW | 0.71** | 0.97±0.24 |  | 0.93** | 1.06±0.26 |  | 0.91^**^ | 0.91±0.22 |
| MMW | 0.86** | 0.90±0.35 |  | 0.86** | 1.07±0.38 |  | 0.78^**^ | 0.77±0.26 |
| HMW | 0.92** | 0.88±0.42 |  | 0.88** | 0.97±0.38 |  | 0.92^**^ | 0.81±0.35 |
| ∑19PAHs | 0.91** | 0.88±0.34 |  | 0.77** | 1.00±0.39 |  | 0.83^**^ | 0.80±0.29 |

Level of significance: *: p < 0.05; **: p< 0.01.

**Table S6** The correlation between PAHs with temperature and wind speed.

|  | Temperature^1^ (n=31) | Temperature^2^ (n=23) | | Wind speed(n=31) |
| --- | --- | --- | --- | --- |
| LMW | 0.18 | | 0.27 | 0.28 |
| MMW | 0.55^**^ | | 0.76^**^ | 0.13 |
| HMW | 0.06 | | 0.44^*^ | 0.06 |
| PAHs | 0.31 | | 0.59^**^ | 0.18 |

Level of significance: **: p< 0.01. 1 represents all data, and 2 represents data with temperatures higher than 20 ℃.

Table S7 TEQ_Bap_ values (ng/m^3^) calculated from PAHs.

| Species | Overall | |  | Summer | |  | Autumn | |
| --- | --- | --- | --- | --- | --- | --- | --- | --- |
|  | Indoor | Outdoor |  | Indoor | Outdoor |  | Indoor | Outdoor |
| NAP | 7.04E-04 | 7.63E-04 |  | 6.27E-04 | 6.05E-04 |  | 7.59E-04 | 8.76E-04 |
| ACY | 2.02E-04 | 2.23E-04 |  | 1.72E-04 | 1.74E-04 |  | 2.24E-04 | 2.55E-04 |
| ACE | 1.94E-04 | 2.08E-04 |  | 1.68E-04 | 1.79E-04 |  | 2.12E-04 | 2.30E-04 |
| FLU | 3.02E-04 | 3.44E-04 |  | 2.69E-04 | 2.84E-04 |  | 3.26E-04 | 3.87E-04 |
| PHE | 8.39E-04 | 9.87E-04 |  | 4.97E-04 | 4.96E-04 |  | 1.09E-03 | 1.34E-03 |
| ANT | 2.19E-03 | 2.33E-03 |  | 1.92E-03 | 1.81E-03 |  | 2.38E-03 | 2.71E-03 |
| FLT | 1.57E-03 | 1.91E-03 |  | 7.64E-04 | 7.30E-04 |  | 2.15E-03 | 2.76E-03 |
| PYR | 1.21E-03 | 1.53E-03 |  | 5.86E-04 | 5.90E-04 |  | 1.67E-03 | 2.22E-03 |
| BaA | 6.95E-02 | 8.95E-02 |  | 4.27E-02 | 4.25E-02 |  | 8.88E-02 | 1.23E-01 |
| CHR | 1.23E-02 | 1.76E-02 |  | 6.78E-03 | 7.80E-03 |  | 1.64E-02 | 2.46E-02 |
| BkF | 2.79E-01 | 3.77E-01 |  | 1.34E-01 | 1.51E-01 |  | 3.84E-01 | 5.40E-01 |
| BbF | 8.54E-02 | 1.10E-01 |  | 4.88E-02 | 5.58E-02 |  | 1.12E-01 | 1.50E-01 |
| BeP | 2.89E-03 | 3.91E-03 |  | 1.43E-03 | 1.71E-03 |  | 3.94E-03 | 5.50E-03 |
| BaP | 1.64E+00 | 2.14E+00 |  | 7.41E-01 | 8.66E-01 |  | 2.29E+00 | 3.05E+00 |
| DahA | 5.05E-01 | 5.93E-01 |  | 3.65E-01 | 4.19E-01 |  | 5.68E-01 | 6.90E-01 |
| IcdP | 1.62E-01 | 2.15E-01 |  | 6.86E-02 | 8.73E-02 |  | 2.30E-01 | 3.07E-01 |
| BghiP | 1.69E-02 | 2.21E-02 |  | 7.34E-03 | 9.49E-03 |  | 2.39E-02 | 3.13E-02 |
| ∑17PAHs | 2.78 | 3.57 |  | 1.42 | 1.65 |  | 3.72 | 4.93 |

Table S8 HI values of PAHs by aged-specific groups.

|  |  | **Childhood (1-11)** | |  | **Adolescence (12-17)** | |  | **Adulthood (18-70)** | |
| --- | --- | --- | --- | --- | --- | --- | --- | --- | --- |
|  |  | **Male** | **Female** |  | **Male** | **Female** |  | **Male** | **Female** |
|  | NAP | 1.5E-06 | 1.56E-06 |  | 1.99E-06 | 2.09E-06 |  | 4.01E-06 | 3.67E-06 |
|  | ACE | 1.37E-07 | 1.43E-07 |  | 1.82E-07 | 1.92E-07 |  | 3.68E-07 | 3.37E-07 |
|  | FLU | 3.25E-07 | 3.39E-07 |  | 4.32E-07 | 4.54E-07 |  | 8.71E-07 | 7.97E-07 |
|  | PHE | 2.42E-06 | 2.53E-06 |  | 3.22E-06 | 3.39E-06 |  | 6.5E-06 | 5.95E-06 |
| Overall | ANT | 3.09E-08 | 3.22E-08 |  | 4.11E-08 | 4.32E-08 |  | 8.28E-08 | 7.58E-08 |
|  | FLT | 1.71E-06 | 1.78E-06 |  | 2.27E-06 | 2.39E-06 |  | 4.59E-06 | 4.2E-06 |
|  | PYR | 3.56E-06 | 3.71E-06 |  | 4.73E-06 | 4.98E-06 |  | 9.55E-06 | 8.74E-06 |
|  | BaP | 2.43E-04 | 2.53E-04 |  | 3.22E-04 | 3.39E-04 |  | 6.50E-04 | 5.95E-04 |
|  | BghiP | 5.02E-06 | 5.23E-06 |  | 6.67E-06 | 7.01E-06 |  | 1.35E-05 | 1.23E-05 |
|  | HI_Total_ | 2.57E-04 | 2.68E-04 |  | 3.42E-04 | 3.59E-04 |  | 6.90E-04 | 6.31E-04 |
|  | NAP | 1.3E-06 | 1.36E-06 |  | 1.73E-06 | 1.82E-06 |  | 3.49E-06 | 3.2E-06 |
|  | ACE | 1.19E-07 | 1.24E-07 |  | 1.58E-07 | 1.66E-07 |  | 3.19E-07 | 2.92E-07 |
|  | FLU | 2.84E-07 | 2.96E-07 |  | 3.78E-07 | 3.97E-07 |  | 7.63E-07 | 6.98E-07 |
| Summer | PHE | 1.39E-06 | 1.45E-06 |  | 1.84E-06 | 1.94E-06 |  | 3.72E-06 | 3.4E-06 |
|  | ANT | 2.64E-08 | 2.76E-08 |  | 3.51E-08 | 3.69E-08 |  | 7.09E-08 | 6.49E-08 |
|  | FLT | 7.93E-07 | 8.27E-07 |  | 1.05E-06 | 1.11E-06 |  | 2.13E-06 | 1.95E-06 |
|  | PYR | 1.64E-06 | 1.71E-06 |  | 2.17E-06 | 2.29E-06 |  | 4.39E-06 | 4.02E-06 |
|  | BaP | 1.07E-04 | 1.11E-04 |  | 1.42E-04 | 1.49E-04 |  | 2.87E-04 | 2.62E-04 |
|  | BghiP | 2.17E-06 | 2.26E-06 |  | 2.88E-06 | 3.03E-06 |  | 5.81E-06 | 5.32E-06 |
|  | HI_Total_ | 1.15E-04 | 1.19E-04 |  | 1.52E-04 | 1.60E-04 |  | 3.07E-04 | 2.81E-04 |
|  | NAP | 1.64E-06 | 1.71E-06 |  | 2.18E-06 | 2.29E-06 |  | 4.39E-06 | 4.02E-06 |
|  | ACE | 1.50E-07 | 1.57E-07 |  | 2.00E-07 | 2.10E-07 |  | 4.03E-07 | 3.69E-07 |
|  | FLU | 3.54E-07 | 3.69E-07 |  | 4.70E-07 | 4.95E-07 |  | 9.49E-07 | 8.69E-07 |
|  | PHE | 3.17E-06 | 3.31E-06 |  | 4.22E-06 | 4.43E-06 |  | 8.51E-06 | 7.79E-06 |
| Autumn | ANT | 3.41E-08 | 3.56E-08 |  | 4.53E-08 | 4.77E-08 |  | 9.15E-08 | 8.37E-08 |
|  | FLT | 2.38E-06 | 2.48E-06 |  | 3.16E-06 | 3.32E-06 |  | 6.37E-06 | 5.83E-06 |
|  | PYR | 4.95E-06 | 5.16E-06 |  | 6.58E-06 | 6.92E-06 |  | 1.33E-05 | 1.22E-05 |
|  | BaP | 3.41E-04 | 3.55E-04 |  | 4.52E-04 | 4.76E-04 |  | 9.13E-04 | 8.36E-04 |
|  | BghiP | 7.08E-06 | 7.38E-06 |  | 9.40E-06 | 9.89E-06 |  | 1.90E-05 | 1.74E-05 |
|  | HI_Total_ | 3.60E-04 | 3.76E-04 |  | 4.79E-04 | 5.03E-04 |  | 9.66E-04 | 8.84E-04 |

Table S9 ILCR values of PAHs by aged-specific groups.

|  | **Childhood (1-11)** | |  | **Adolescence (12-17)** | |  | **Adulthood (18-70)** | |
| --- | --- | --- | --- | --- | --- | --- | --- | --- |
|  | **Male** | **Female** |  | **Male** | **Female** |  | **Male** | **Female** |
| **ILCR_Inhalation_** | | | | | | | | |
| **Summer** | 1.09E-10 | 1.12E-10 |  | 2.02E-10 | 2.09E-10 |  | 4.69E-10 | 4.12E-10 |
| **Autumn** | 2.94E-10 | 3.02E-10 |  | 5.47E-10 | 5.65E-10 |  | 1.27E-09 | 1.11E-09 |
| **Overall** | 2.18E-10 | 2.24E-10 |  | 4.05E-10 | 4.19E-10 |  | 9.40E-10 | 8.25E-10 |
| **ILCR_Ingestion_** | | | | | | | | |
| **Summer** | 6.68E-06 | 6.87E-06 |  | 7.96E-06 | 8.23E-06 |  | 6.47E-06 | 7.03E-06 |
| **Autumn** | 1.81E-05 | 1.86E-05 |  | 2.15E-05 | 2.23E-05 |  | 1.75E-05 | 1.90E-05 |
| **Overall** | 1.34E-05 | 1.38E-05 |  | 1.60E-05 | 1.65E-05 |  | 1.30E-05 | 1.41E-05 |
| **ILCR_Dermal_** | | | | | | | | |
| **Summer** | 8.33E-06 | 8.56E-06 |  | 9.93E-06 | 1.03E-05 |  | 1.15E-05 | 1.25E-05 |
| **Autumn** | 2.25E-05 | 2.31E-05 |  | 2.68E-05 | 2.77E-05 |  | 3.10E-05 | 3.37E-05 |
| **Overall** | 1.67E-05 | 1.72E-05 |  | 1.99E-05 | 2.06E-05 |  | 2.30E-05 | 2.50E-05 |
| **ILCR_Total_** | | | | | | | | |
| **Summer** | 1.50E-05 | 1.54E-05 |  | 1.79E-05 | 1.85E-05 |  | 1.80E-05 | 1.95E-05 |
| **Autumn** | 4.06E-05 | 4.17E-05 |  | 4.84E-05 | 5.00E-05 |  | 4.85E-05 | 5.27E-05 |
| **Overall** | 3.01E-05 | 3.09E-05 |  | 3.59E-05 | 3.71E-05 |  | 3.60E-05 | 3.91E-05 |

**References:**

Ali-Taleshi, M.S., Moeinaddini, M., Bakhtiari, A.R., Feiznia, S., Squizzato, S., Bourliva A., 2021. A one-year monitoring of spatiotemporal variations of PM_2.5_-bound PAHs in Tehran, Iran: Source apportionment, local and regional sources origins and source-speciﬁc cancer risk assessment. Environ. Pollut., 274, 115883.

Błaszczyk, E., Rogula-Kozłowska, W., Klejnowski, K., Fulara, I., Mielżyńska-Švach, D., 2017. Polycyclic aromatic hydrocarbons bound to outdoor and indoor airborne particles (PM_2.5_) and their mutagenicity and carcinogenicity in Silesian kindergartens, Poland. Air Qual. Atmos. Health, 10, 389–400.

Chen, Y., Li, X.H., Zhu, T.L., Han, Y.J., Lv, D., 2017. PM_2.5_-bound PAHs in three indoor and one outdoor air in Beijing: Concentration, source and health risk assessment. Sci. Total Environ., 586, 255–264.

Chen, Y.T., Lai, B., Wei, Y.X., Ma, Q.W., Liang, H.R., Yang, H., 2022. Polluting characteristics, sources, cancer risk, and cellular toxicity of PAHs bound in atmospheric particulates sampled from an economic transformation demonstration area of Dongguan in the Pearl River Delta, China. Environ. Res., 215, 114383.

Han, Y.J., Li, X.H., Zhu, T.L., Lv, D., Chen, Y., Hou, L.A., Zhang, Y.P., Ren, M.Z., 2016. Characteristics and relationships between indoor and outdoor PM_2.5_ in Beijing: a residential apartment case study. Aerosol Air Qual. Res., 16, 2386–2395.

Hong, Y.W., Xu, X.B., Liao, D., Ji, X.T., Hong, Z.Y., Chen, Y.T., Xu, L.L., Li, M.R., Wang, H., Zhang, H., Xiao, H., Choi, S.D., Chen, J.S., 2021. Air pollution increases human health risks of PM_2.5_-bound PAHs and nitro-PAHs in the Yangtze River Delta, China. Sci. Total Environ., 770, 145402.

Kalisa, E., Nagato, E., Bizuru, E., Lee, K., Tang, N., Pointing, Stephen., 2019. Pollution characteristics and risk assessment of ambient PM_2.5_-bound PAHs and NPAHs in typical Japanese and New Zealand cities and rural sites. Atmos. Pollut. Res., 10, 1396–1403.

Li, Y.Y., Yang, L.X., Chen, X.F., Gao, Y., Jiang, P., Zhang, J.M., Yu, H., Wang, W.X., 2017. PM_2.5_-bound PAHs in indoor and outdoor of hotels in urban and suburban of Jinan, China: concentrations, sources, and health risk impacts. Aerosol Air Qual. Res., 17, 2463–2473.

Li, Y.F., Bai, X.R., Ren, Y.Q., Gao, R., Ji, Y.Y., Wang, Y.F., Li, H., 2022. PAHs and nitro-PAHs in urban Beijing from 2017 to 2018: Characteristics, sources, transformation mechanism and risk assessment. J. Hazard Mater., 436, 129143.

Romagnoli, P., Perilli, M., Gordiani, A., Gatto, M.P., Cecinato, A., 2014. Indoor PAHs at schools, homes and ofﬁces in Rome, Italy. Atmos. Environ., 92, 51–59.

Ting, Y.C., Zou, Y.X., Pan, S.Y., Ko, Y.R., Ciou, A.J., Huang, C.H., 2024. Sources-attributed contributions to health risks associated with PM_2.5_-bound polycyclic aromatic hydrocarbons during the warm and cold seasons in an urban area of Eastern Asia. Sci. Total Environ., 922, 171325.

Wang, L.J., Dong, S.Z., Liu, M.M., Tao, W.D., Xiao, B., Zhang, S.W., Zhang, R.Q., Li, X.P., 2019b. Polycyclic aromatic hydrocarbons in atmospheric PM_2.5_ and PM_10_ in the semi-arid city of Xi'an, Northwest China: Seasonal variations, sources, health risks, and relationships with meteorological factors. Atmos. Res., 229, 60–73.

Zhang, J.M., Yang, L.X., Mellouki, A., Chen, J.M., Chen, X.F., Gao, Y., Jiang, P., Li, Y.Y., Yu, H., Wang, W.X., 2018. Diurnal concentrations, sources, and cancer risk assessments of PM_2.5_-bound PAHs, NPAHs, and OPAHs in urban, marine and mountain environments. Chemosphere, 209, 147–155.

Zhang, Y., Yang, L.X., Zhang, X.F., Li, J.S., Zhao, T., Gao, Y., 2019a. Characteristics of PM_2.5_-bound PAHs at an Urban Site and a Suburban Site in Jinan in North China Plain. Aerosol Air Qual. Res., 19, 871–884.

Zhang, Y., Zheng, H., Zhang, L., Zhang, Z.Z., Xing, X.L., Qi, S.H., 2019b. Fine particle-bound polycyclic aromatic hydrocarbons (PAHs) at an urban site of Wuhan, central China: Characteristics, potential sources and cancer risks apportionment. Environ. Pollut., 246, 319–327.

Zhang, L., Yang, Z., Liu, J.J., Zeng, H., Fang, B., Xu, H.J., Wang, Q., 2021. Indoor/outdoor relationships, signatures, sources, and carcinogenic risk assessment of polycyclic aromatic hydrocarbons-enriched PM_2.5_ in an emerging port of northern China. Environ. Geochem. Health, 43, 3067–3081.

Zhu, Y.H., Yang, L.X., Meng, C.P., Yuan, Q., Yan, C., Dong, C., Sui, X., Yao, L., Yang, F., Lu, Y.L., Wang, W.X., 2015. Indoor/outdoor relationships and diurnal/nocturnal variations in water-soluble ion and PAH concentrations in the atmospheric PM_2.5_ of a business ofﬁce area in Jinan, a heavily polluted city in China. Atmos. Res., 153, 276–285.
